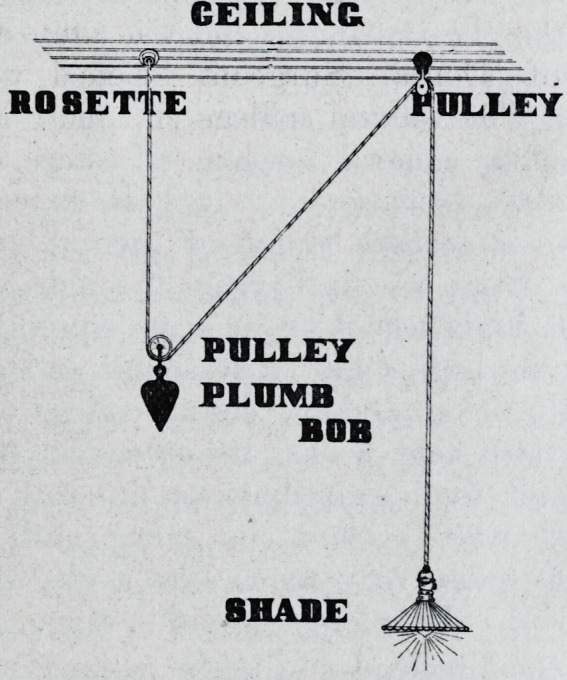# A Handy Electric Light

**Published:** 1904-09

**Authors:** L. O. Frantz

**Affiliations:** Alliance, O.


### A HANDY ELECTRIC LIGHT.

By L. O. Frantz, D. D. S., Alliance, O.

(Written for *The American Journal of Dental Science.*)

Get two small awning pulleys costing five cents each and a plumb bob weighing one or two pounds according to the weight of the lamp and shade. Have the lamp cord long enough to reach from the ceiling to the floor and a foot extra or so that it will reach to any part of the laboratory unless it is a large laboratory then you will need several fixed this way. Run wire from rosett on ceiling through one pulley with plumb bob wired fast as in cut then through the other pulley fastened to ceiling then to lamp. Cut explains the article.

In my laboratory which is five feet by eight feet with benches around the sides one lamp is sufficient for all purposes. A cheap shade is made from a five cent funnel with small end unsoldered and slits an inch long cut down the sides and bent around the socket with a few strands of binding wire wrapped around the ends to hold them in place. Care should be taken not to get ends of wire connected if current is on at time as the fuse wire in rosett will be blown out; better unscrew rosett cover.

Plumb bob costs from ten cents to twenty cents in any hardware store.

### TO LINE ARTIFICIAL DENTURES WITH ALUMINUM.

By Clint W. La Salle, D. D. S., Rochester, N. Y.

(Written for *The American Journal of Dental Science.*)

## Figures and Tables

**Figure f1:**